# Habitat corridors facilitate genetic resilience irrespective of species dispersal abilities or population sizes

**DOI:** 10.1111/eva.12255

**Published:** 2015-03-31

**Authors:** Mark R Christie, L Lacey Knowles

**Affiliations:** 1 Ecology and Evolutionary Biology, University of MichiganAnn Arbor, MI, USA; 2 Department of Biological Sciences & Department of Forestry and Natural Resources, Purdue UniversityWest Lafayette, IN, USA

**Keywords:** corridor, dispersal, gene flow, genetic diversity, genetic resilience, habitat fragmentation, species interactions

## Abstract

Corridors are frequently proposed to connect patches of habitat that have become isolated due to human-mediated alterations to the landscape. While it is understood that corridors can facilitate dispersal between patches, it remains unknown whether corridors can mitigate the negative genetic effects for entire communities modified by habitat fragmentation. These negative genetic effects, which include reduced genetic diversity, limit the potential for populations to respond to selective agents such as disease epidemics and global climate change. We provide clear evidence from a forward-time, agent-based model (ABM) that corridors can facilitate genetic resilience in fragmented habitats across a broad range of species dispersal abilities and population sizes. Our results demonstrate that even modest increases in corridor width decreased the genetic differentiation between patches and increased the genetic diversity and effective population size within patches. Furthermore, we document a trade-off between corridor quality and corridor design whereby populations connected by high-quality habitat (i.e., low corridor mortality) are more resilient to suboptimal corridor design (e.g., long and narrow corridors). The ABM also revealed that species interactions can play a greater role than corridor design in shaping the genetic responses of populations to corridors. These results demonstrate how corridors can provide long-term conservation benefits that extend beyond targeted taxa and scale up to entire communities irrespective of species dispersal abilities or population sizes.

## Introduction

Agriculture and urbanization currently account for 43% of the earth's land area (Vitousek et al. 1997; Foley et al. 2011; Barnosky et al. 2012), and much of the remaining area is interlaced with networks of roads (Barnosky et al. 2012). These substantial disturbances to natural ecosystems and communities are projected to continue (Haberl et al. 2007; Foley et al. 2011) and have created a conspicuous mosaic of habitat patches embedded within a matrix of altered landscapes. This habitat fragmentation subdivides populations and can profoundly alter both ecological and evolutionary dynamics at population, species, and community-wide levels (Hanski 1994, 1999; Tewksbury et al. 2002). One approach to preventing or restoring subdivided populations is to connect fragmented habitat patches with corridors (Beier and Noss 1998; Brudvig et al. 2009; Gilbert-Norton et al. 2010). Such corridors have been well studied from an ecological perspective (Gilbert-Norton et al. 2010), but little work has examined the genetic effects of corridors that connect habitat patches. The evaluation of evolutionary forces such as genetic drift and gene flow is critical for determining the long-term effects of habitat fragmentation; any resulting genetic changes will affect how populations respond to current and future agents of selection such as increased disease risk, invasive species, and global climate change (Stockwell et al. 2003; Hughes and Boomsma 2004; Spielman et al. 2004; Doi et al. 2010; Pauls et al. 2013).

Both the optimal design and the realized benefits of corridors have been the focus of considerable debate (Mann and Plummer 1995; Haddad and Tewksbury 2005; Gilbert-Norton et al. 2010; Beier and Gregory 2012). Determining the effectiveness of corridors can be controversial because the detection of positive or negative effects is subject to the response variables being measured, the specific ecological context, and the individual species being studied (Haddad and Tewksbury 2005; Gregory and Beier 2014). In general, however, ecological studies have found that corridors can facilitate movement between patches and can increase species richness and abundance (Brudvig et al. 2009; Gilbert-Norton et al. 2010). It has also been shown that corridor design, which can vary substantially between locations, can influence dispersal behavior, migration rates, and community composition (Harrison 1992; Lindenmayer and Nix 1993; Andreassen et al. 1996; Haddad 1999; Damschen et al. 2008). It is therefore likely that corridors and their design will also have a substantial effect on the genetics of populations. Furthermore, it is well known that habitat fragmentation can result in increased genetic drift and/or inbreeding, which can reduce effective population sizes and genetic diversity (Alo and Turner 2005; Aguilar et al. 2008; Mendez et al. 2014). Whether corridors can reduce these negative genetic effects remains largely untested. Limited empirical data have shown that, for some species, corridors can increase gene flow between patches (Mech and Hallett 2001; Sharma et al. 2013). However, it can be challenging to discriminate between low and moderate amounts of gene flow, particularly in subdivided populations (Waples 1998). Thus, there is a growing need not only to understand whether corridors can mitigate the negative genetic effects of habitat fragmentation, but also to gain a mechanistic understanding toward corridor design and the resulting eco-evolutionary dynamics.

Beyond the spatial design of corridors, one factor that may regulate connectivity between fragmented habitats is corridor quality (Haddad and Tewksbury 2005). If corridor quality is low, then some species may not successfully disperse between patches due to behavioral inhibition or increased mortality in corridors. Species interactions may also play an important role in determining corridor quality as succession, priority effects, and community assemblage theory may dictate which species persist in a corridor. Furthermore, it has been shown that species interactions can influence dispersal distances and population sizes (Tilman and Kareiva 1997; McPeek and Peckarsky 1998; Fagan et al. 1999; Boulangeat et al. 2012; Ruokolainen and Ripa 2012; Urban et al. 2013), which could result in downstream genetic effects within a patch–corridor framework. Because species interactions can range from strongly negative to strongly positive and can vary depending on the focal species, a comprehensive understanding of species interactions may assist corridor implementation. Yet, to our knowledge, there are no studies that have examined the genetic effects of corridors from a community-level perspective. Instead, a large percentage of corridors are targeted toward single species, even though the focal species may be best served by a patch–corridor environment that accounts for the evolutionary and conservation potential of the entire community (Haddad et al. 2003).

**Figure 1:**
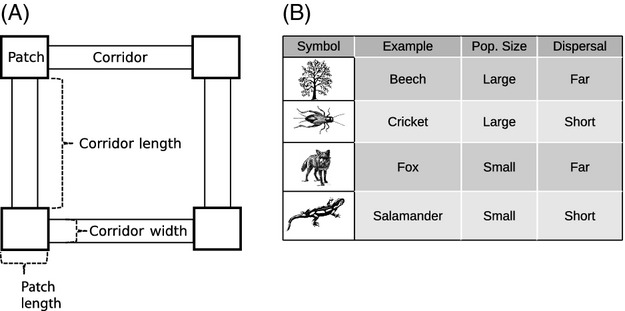
Illustration of model design and species groups. Panel A illustrates the basic design where each patch was connected by two corridors. We varied corridor length and corridor width to examine the effects of different corridor–habitat configurations. Panel B illustrates the four species groups we modeled and their corresponding symbols, population sizes, and dispersal abilities (see Materials and methods for details).

To test for the genetic effects of habitat fragmentation with corridors, we constructed a forward-time, agent-based model that tracks individuals and their associated genotypes through space and time. Here, we model a patch–corridor system that consists of patches of naturally occurring or restored habitat that are connected by comparatively thin strips of habitat (i.e., corridors). We constructed habitat patches connected by corridors where we could vary the size of the patches and the length and width of the corridors (Fig.1A). The results from this model are generally applicable to any system where habitat patches are embedded in a matrix with different habitat characteristics (e.g., decreased survival) and where the patches can be connected with corridors. We created four species groups characterized by differing population sizes and dispersal distances (Fig.1B), two ecological characteristics that can translate to differing amounts of genetic drift and gene flow (Hedrick 2005; Allendorf et al. 2013). The parameters characterizing the species groups were chosen to encompass a variety of taxa such that the qualitative results remain unchanged regardless of actual population sizes or dispersal distances that were examined. We first examined how corridor width and corridor length affect the genetic diversity, genetic differentiation, and effective population sizes of individuals occupying habitat patches. Both genetic diversity and genetic differentiation are important response variables because genetic diversity can determine the future potential of a population to respond to a changing environment (Allendorf et al. 2013), and genetic differentiation is dictated by the amount of genetic drift within and gene flow between habitat patches (Frankham et al. 2002). We also examined the effects of corridor quality by varying the rates of mortality within the corridors. Lastly, we identified how both positive and negative species interactions can affect both genetic diversity and genetic differentiation. We conclude by identifying general properties of patch–corridor systems that can influence the genetic resilience of entire communities irrespective of species dispersal abilities or population sizes.

## Materials and methods

### Model development

To test for the genetic effects of different habitat corridors, we developed a forward-time, agent-based model that tracked individuals and their associated genotypes through space and time. The default model setup consisted of four patches connected by four habitat corridors embedded in a matrix (Fig.1A). Population size was varied between small (*n* = 500) and large (*n* = 1000) and represented the total carrying capacity across all four patches. We chose these population sizes because they reflected a balance between being small enough to remain important from a conservation perspective, but large enough to avoid immediate extinction. We further varied population sizes beyond these two values with little change to qualitative patterns (see Model sensitivity). Carrying capacity in the corridors was proportional to the total corridor area, and corridor population density was set equal to patch density. Each model run was initiated with the placement of habitat patches and corridors in a Cartesian plane. Equal numbers of individuals were distributed randomly throughout each patch, and each individual was characterized by 50 bi-allelic loci with a minor allele frequency of 0.2. Diploid genotypes were created for each individual in accordance with Hardy–Weinberg equilibrium. At the beginning of each trial, allele frequencies were equal between patches (i.e., *F*_ST_ = 0) to simulate the fragmentation of a single population. Reproduction was asexual, with each individual producing 20 propagules. Sexual reproduction was not modeled because the mechanism depends upon behavior (i.e., asexual reproduction is useful as a null model) (Martins et al. 2013). There are a couple of reasons why modeling asexual reproduction is useful as null model. First, when including sexual reproduction, one must choose a maximum allowable distance between two individuals to let them reproduce. This distance is somewhat arbitrary and, more importantly, would constrain individuals from dispersing successfully (i.e., if a solitary individual dispersed down a corridor, but could not find a mate, then it would be removed in the next generation). Secondly, in patches with small or declining population sizes, individuals may not be able to find mates and the patch could become extinct more quickly. Because we varied both dispersal and population sizes directly, adding reproduction to the model would not yield additional insights (see Discussion for details regarding the effects of behavior).

Dispersal was modeled as a multivariate normal distribution with mean displacement equals to zero. Thus, dispersal was passive and unbiased with respect to direction. Species dispersed both ‘far’ and ‘short’ distances, where ‘short’ was modeled with a covariance matrix that resulted in 70% less displacement. Increasing or decreasing the variance associated with the two-dimensional dispersal function resulted in greater or lesser amounts of gene flow, respectively. Dispersal distance and direction was determined by sampling from the multivariate normal distribution. Individuals were determined to have dispersed to an area within a patch or a habitat corridor using a modified ray-casting algorithm (Yang and Stern 2013). Individuals that dispersed outside of a patch or corridor (i.e., landed in the matrix) were eliminated from further analysis. If there were more surviving individuals in the patches than dictated by the species carrying capacity, then individuals were randomly removed until the set carrying capacity was obtained. Similarly, if there were more individuals present in the corridors than allowed by the density–area relationship, then individuals were randomly removed until carrying capacity was obtained. Note that variance in reproductive success occurred naturally in this model because of the variable dispersal and survival outcomes. The process of reproduction, dispersal, matrix-associated mortality, and carrying-capacity-associated mortality was repeated for 70 generations. At these ecologically relevant timescales, the effect of even high mutation rates (e.g., 10^−5^) was undetectable. From a management perspective, the short time frame means that the sampling of pre-existing variation, not the introduction of new mutations, is the relevant dynamic for measuring the consequences of corridors on genetic diversity and differentiation.

After a single model run completed, all individuals within each patch were sampled. Genetic diversity was calculated as the number of unique multilocus genotypes across all patches. Because we used 50 bi-allelic diploid loci, the maximum number of unique multilocus genotypes equaled 3^50^, but the actual number at the start of a run was dictated by sample sizes (i.e., the realized number of unique multilocus genotypes could not be greater than the sample size). Genetic differentiation was calculated using an unbiased estimator for *F*_ST_ (Wier and Cockerham 1984). We also implemented two basic model types: one where the total area of the patches was held constant, but the area of the corridors was allowed to vary freely and one where the total area of the patches and corridors was held constant such that any increases in corridor area resulted in a decrease in patch area (and vice versa). All model iterations were implemented in R 3.0.2 with the mnormt, plotrix, and hierfstat packages (Goudet 2005; Lemon 2006; Genz and Azzalini 2013; R core team 2014).

### Corridor design

We first tested the effect of corridor design on the genetic responses of the four species groups by varying corridor width and length. Both corridor width and length were measured in ‘patch lengths’, which represents the length of one side of the square patches (Fig.1A). This unitless property of the model is desirable because the results can be scaled to any distance metrics making the results generalizable to a large number of ecological communities and management scenarios (e.g., urban versus agricultural landscapes). Corridor width was varied from 0.1 to 0.9 in increments of 0.2. Corridor length was varied from 1.0 to 6.0 in increments of 0.5. No mortality was initially imposed in the corridors, such that the density of individuals per unit area of the corridors was equal to that of the patches. We ran 30 replicates for each of the 220 combinations of corridor length, corridor width, and species group. For measures of genetic diversity, we fit a full linear model with all interactions. We fit the linear model to the mean value of the 30 replicates to avoid challenges associated with fitting statistical models to simulated data (White et al. 2014). For measures of genetic differentiation, we fit a thin-plate spline surface to generate contour plots. For fixed corridor lengths, we also examined the effect of corridor width on effective population size. Effective population size was calculated using a linkage disequilibrium method (Do et al. 2014), although qualitative results did not change with the alternative single-sample methods implemented in the program.

### Corridor quality

Because empirical studies have found that corridors can vary in quality (Haddad and Tewksbury 2005; Lees and Peres 2008), we next varied the quality of the corridors by imposing higher rates of mortality in the corridors than in the patches. After the dispersal and carrying-capacity steps, we identified all individuals that had dispersed into a corridor and increased mortality by randomly removing 25%, 50%, 75%, 90%, and 100% of the individuals. We next calculated the change in genetic differentiation and genetic diversity that occurred due to this mortality. This change was measured relative to the model without increased corridor mortality and visualized with heat maps (Warnes et al. 2014). Population sizes in the patches were unaffected as were any individuals that were able to disperse between two patches within a single generation (e.g., at short corridor lengths). Similar to the scenarios without increased corridor mortality, we ran 30 iterations for each combination of corridor width, corridor length, species group, and percent mortality.

### Species interactions

Species interactions were modeled as positive, negative, or neutral (i.e., no effect). To model positive species interactions, we increased population size by 20%, increased the absolute value of the mean of the dispersal kernel by 20%, and reduced corridor mortality by 20% for the focal species (For examples see Tilman and Kareiva 1997; McPeek and Peckarsky 1998; Fagan et al. 1999; Boulangeat et al. 2012; Ruokolainen and Ripa 2012; Urban et al. 2013). To model negative species interactions, we decreased population size by 20%, decreased the absolute value of the mean of the dispersal kernel by 20%, and increased corridor mortality by 20%. Neutral interactions were simulated with the basic model without corridor mortality (described above).

### Model sensitivity

We examined the effect of corridor configuration by arranging the patches in a linear fashion such that the two middle patches were connected by two corridors and the two end patches were only connected by one corridor. In this scenario, the population density of each patch had to be independently regulated to prevent extinction in the two end patches. In the preceding scenarios, where each patch was connected by two corridors, the patches could be regulated at the patch or meta-patch (i.e., all patches) level and no differences in extinction–recolonization rates or genetic response variables were observed. We also measured the effect of fecundity by varying the number of propagules produced by each individual (*n* = 1, 2, 10, 50), and we varied population sizes (*n* = 100, 2000, 5000) and dispersal distances (and dispersal kernel shapes) beyond the four ‘species groups’ to establish that the results remained qualitatively identical. We also examined the effect of running the model for 50, 75, and 100 generations. Modifying all of these parameters (i.e., corridor configuration, number of propagules, population sizes, dispersal parameters, and number of generations) resulted in quantitative but not qualitative changes to the presented analyses.

## Results

**Figure 2:**
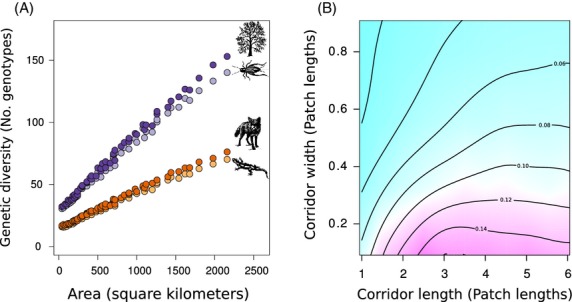
Effect of corridor length and corridor width on genetic diversity (Panel A) and genetic differentiation (Panel B). Genetic diversity in the patches increased linearly with increases in corridor area for all species groups (Panel A). Here, corridor area was measured in square kilometers, but the dimensionless design of the model means that these results can scale to any system (see Materials and methods). Notice that species' population sizes, but not dispersal abilities, are important predictors of genetic diversity and that this effect is amplified at increased corridor areas. Genetic differentiation does not increase linearly with corridor area but rather depends on the specific combination of corridor length and width (Panel B). This panel illustrates the effects for species with small population sizes and short dispersal distances and the contour lines and colors indicate values of genetic differentiation between the patches with blue representing low *F*_ST_ values and red representing high *F*_ST_ values. The precise effects vary between the species groups (see Fig. S2), but for all species, even modest increases in corridor width can result in large reductions in genetic differentiation.

For all four species groups, genetic diversity of populations occupying a habitat patch increased as the corridor area connecting patches increased (Fig.2A; *R*^2^ = 0.59, *P* < 0.001). The mostly linear relationship illustrates that corridor length and width contribute proportionately to genetic diversity within patches (Fig. S1). Across species, population size had a substantial effect on genetic diversity (*R*^2^ = 0.34, *P* < 0.001), but dispersal distance did not (*R*^2^ = 0.002, *P* = 0.882), for the given geographic scales examined. The effect of population size on genetic diversity was greater at large corridor areas than at small corridor areas (i.e., there was a significant interaction between corridor area and population size; *P* < 0.001).

In contrast to genetic diversity, corridor length and width did not contribute proportionately to the accumulation of genetic differentiation between individuals occupying patches (Figs2B and S2). Regardless of corridor length, increased corridor width resulted in lower amounts of genetic differentiation among patches. Furthermore, when corridors are not wide relative to the dimensions of the patch, even small increases in corridor width can result in a substantial decrease in genetic differentiation. At small distances between patches, corridor length is important because even slight increases in the length of the corridor can result in fewer individuals dispersing between patches. At larger corridor lengths, the effect of corridor length is negligible because slightly shorter or longer corridors will still not facilitate dispersal between patches over short time periods. As expected, species with small population sizes that dispersed short distances had greater overall levels of genetic differentiation than did species with large population sizes that dispersed greater distances (Fig. S3).

**Figure 3:**
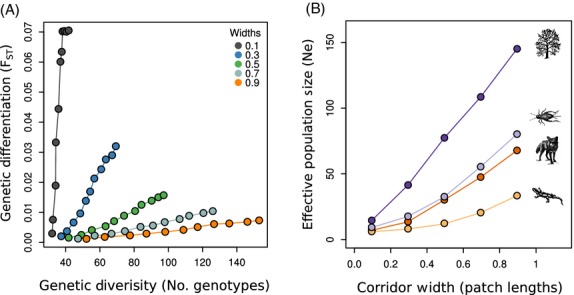
Panel A illustrates the relationship between genetic diversity and genetic differentiation with different corridor designs. For a given corridor width (shown as different colors), each increase along the *x* and *y* axes (line segments) corresponds to an increase in corridor length. Even small increases in corridor width can result in substantial decreases in *F*_ST_ between patches with concomitant increases in genetic diversity. This general relationship is maintained even when dispersal between patches is reduced (i.e., irrespective of whether the life history characteristics of a taxon is more similar to a salamander as opposed to a fox). This result occurs because increased corridor width results in decreased variance in reproductive success, lower genetic drift, and higher effective population size in the patches (Panel B). Notice that the effective population size in the patches increases even though the absolute (i.e., census) population size in a patch for each species was held constant.

Regardless of the species characteristics, increased corridor width resulted in substantial reductions in genetic differentiation with concomitant increases in overall levels of genetic diversity within patches (Figs3A and S4). Furthermore, at small corridor widths, even short increases in corridor length can result in substantial increases in genetic differentiation. Conversely, at larger corridor widths, increasing the length of the corridor results in substantial gains in genetic diversity within patches and only small increases in genetic differentiation (Fig.3A). The benefits of increasing corridor width occur because, even if individuals cannot disperse between patches within a single generation, a wide corridor facilitates higher absolute population sizes in the surrounding area, encouraging the exchange of individuals across a broader area, and reducing the variance in reproductive success and subsequent genetic drift (Christie et al. 2012). The joint effect of these processes is an increase in the effective population size within each patch (Fig.3B), despite the census population size in each patch being held constant.

**Figure 4:**
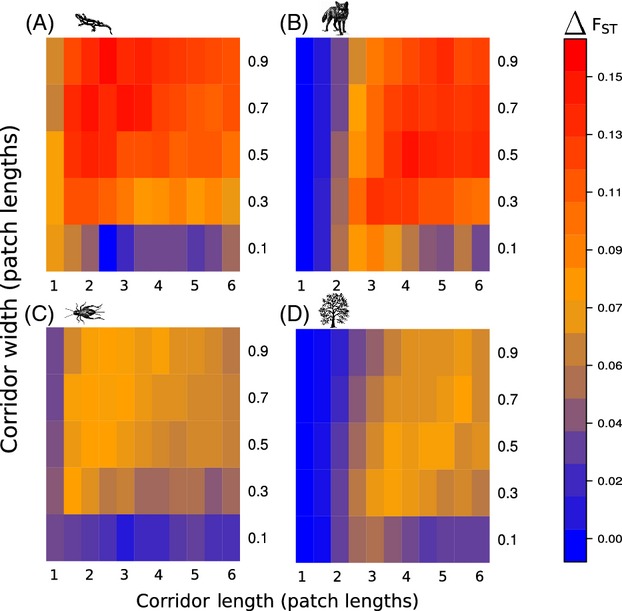
A heat map illustrating the differences in genetic differentiation (*F*_ST_) between scenarios with high corridor mortality and those without increased corridor mortality (Panels A-D). Species with small population sizes experienced the largest increases in genetic differentiation (A and B) when exposed to high corridor mortality. Species with high dispersal distances were not substantially affected by corridor mortality at short corridor lengths (B and D). Notice that in most cases, corridor mortality resulted in an increase in genetic differentiation, which suggests that high-quality habitat corridors (those with low mortality) may be the most useful for mitigating the negative genetic effects of habitat fragmentation.

In most cases, corridor mortality resulted in higher rates of genetic differentiation (Fig.4) and lower levels of genetic diversity (Fig. S5). For the same percentage of corridor mortality, species with smaller population sizes had larger increases in genetic differentiation than did species with larger population sizes (Fig.4). Conversely, species with larger population sizes had larger absolute reductions in genetic diversity than did species with smaller population sizes (Fig. S5). For species that could disperse far distances within a single generation, the effect of corridor mortality was trivial at short distances because individuals continued to disperse between patches. There were also less substantial changes in both genetic differentiation and genetic diversity at narrow corridor widths. This result occurred because patches connected with narrow corridors already had comparatively high rates of genetic differentiation (e.g., Fig.2B), and the increase in mortality did not result in a substantial decrease in the already low migration rates. Lastly, as corridor mortality increases, the benefits associated with increased corridor width decrease and the importance of corridor length increases (Figs S6 and S7). This phenomenon occurs because as corridor mortality increases it becomes more beneficial to disperse to suitable patch habitat rather than to establish in the corridor itself. Nevertheless, even patches joined by long, thin, corridors with very high mortality (e.g., 90%) provided greater genetic resilience to the habitat patches than environments where corridors were entirely absent (Fig. S7).

**Figure 5:**
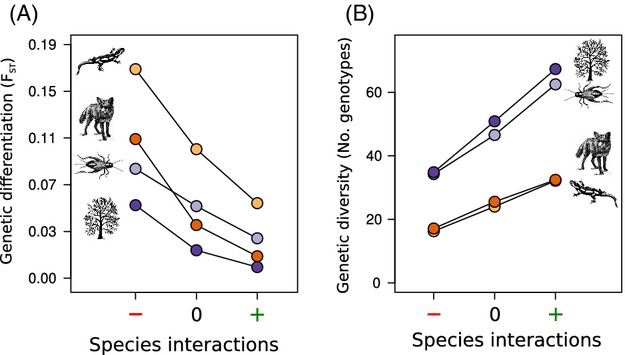
The effects of positive and negative species interactions on genetic differentiation and diversity. Panel A illustrates that, for all species groups, positive species interactions can reduce genetic differentiation between patches, while negative species interactions can increase genetic differentiation. Both population size and dispersal abilities associated with the species play a role in determining the effect size. Panel B illustrates that positive species interactions can increase genetic diversity in the patches, while negative species interactions can decrease genetic diversity. Genetic diversity is much more strongly influenced by a species' population sizes than by their dispersal characteristics.

For all species, positive species interactions (any interaction with a positive effect on the focal species) resulted in lower levels of genetic differentiation, while negative species interactions resulted in higher levels of genetic differentiation between individuals sampled in the patches (Fig.5A). Both population size and dispersal ability were important for predicting the amount of genetic differentiation. For all species, positive species interaction increased the total amount of genetic diversity, while negative species interactions decreased the total amount of genetic diversity (Fig.5B). The relationship between species interactions and genetic diversity was almost entirely a function of population size as opposed to dispersal capability. These results illustrate that it is necessary to account for species interactions when designing corridors and that accounting for dispersal ability is more important for minimizing genetic differentiation than maintaining genetic diversity.

## Discussion

The results presented here demonstrate the mitigating effects of corridors on the negative genetic effects associated with habitat fragmentation are achieved through two mechanisms: the exchange of individuals between patches and the reduction in genetic drift. As a consequence, increasing the width of corridors even by a small percentage can substantially reduce the amount of genetic differentiation between patches while increasing the genetic diversity within patches. These benefits partly occur because increasing corridor width creates higher absolute population sizes in corridor areas surrounding the patches and facilitates the exchange of larger numbers of individuals between habitat patches. However, the benefits associated with increasing corridor width were also documented in (i) models where the carrying capacity of both the patches and the corridors were held constant regardless of the total area and (ii) in species that could not disperse very far distances. Thus, an additional benefit of increasing corridor width is to simply facilitate the survival of offspring from a larger number of individuals, which serves to greatly decrease the inflated variance in reproductive success caused by habitat fragmentation and thus decrease the amount of genetic drift that would otherwise occur. The additional benefits of decreased genetic drift within habitat patches provide additional support for the idea that habitat corridors can be an effective conservation and management tool. Previous theoretical work has suggested that increased gene flow provided by corridors is beneficial (Harrison 1992; Orrock 2005), but this agent-based model demonstrates that both increased gene flow and decreased genetic drift afforded by corridors can synergistically mitigate the negative genetic effects of habitat fragmentation.

The benefits associated with increased corridor width are maximized when corridor habitat quality is high. If corridor quality is low (i.e., mortality in the corridors is high), then poorly dispersing species are not be able to traverse between habitat patches to maximize gene flow or persist in the corridors long enough to reduce the effects of genetic drift in the patches. Thus, we recommend that managers connect habitat patches with corridors that are as wide as possible, but caution that if the quality of the corridors differs substantially from the habitat patches, then some of the potential mitigating effects of corridors may be lost. Nevertheless, even long and narrow corridors with low-quality habitat provided populations with greater genetic resilience than habitat patches that lacked corridors entirely (Fig. S7). Furthermore, there is an interaction between corridor design and corridor quality: populations connected by corridors that are long and thin respond less severely to increases in corridor mortality because the genetic resilience has already been partly eroded. One interesting consequence of this phenomenon is that populations connected by wide corridors are more resilient to increases in corridor mortality (i.e., poor-quality corridor habitat), whereas populations connected by high-quality habitat (i.e., low corridor mortality) are more resilient to suboptimal corridor design (e.g., long and narrow).

Species interactions can also play a substantial role in shaping the genetic responses of populations to habitat corridors. Here, we show that positive species interactions can further mitigate the negative genetic effects of habitat fragmentation when corridors are present, while negative species interactions can increase genetic differentiation and reduce genetic diversity. These results highlight the importance of designing corridors for entire communities and not focusing efforts on single species because key ecological principles (e.g., succession, facilitation) can translate into substantial evolutionary consequences. From a conservation and management perspective, it will be most useful to quantify the magnitude of species interactions that influence species dispersal, persistence, and reproduction in habitat corridors (Berlow et al. 1999). Moreover, by accounting for species interactions, corridors designed for entire communities may provide greater benefits to single targeted species.

Animal behavior and matrix quality are two system-specific characteristics worth further consideration. Because dispersal behavior can be multifaceted and difficult to predict, we did not include it in this genetic approach. For some species, it is possible that behavior could facilitate dispersal between patches, including compensatory movement through low-quality habitat (Haddad and Tewksbury 2005). However, for other species, behavior may also limit dispersal between patches (LaPoint et al. 2013). Thus, the effects of behavior are difficult to predict and may warrant study on a case-by-case basis (Haddad 1999). While behavior may serve to increase or decrease dispersal rates between patches, the qualitative results presented here would remain unchanged. Likewise, all else being equal, reduced mortality within the matrix may provide some of the positive benefits associated with increasing corridor width (e.g., increases in the effective population sizes of patches), provided that individuals inhabiting the matrix could contribute progeny back to corridors and habitat patches (Pulliam 1988; Hanski 1994). In this way, portions of the matrix could act as additional habitat patches for species that could disperse easily without corridors (Ricketts 2001).

Local adaptation and the evolutionary implications of genetic differentiation also warrant further consideration. If there is strong selection specific to each habitat patch that promotes local adaptation, then immigrants from other patches will experience decreased survival and reduced reproductive success (Peterson et al. 2014; Somervuo et al. 2014). Thus, even though dispersal between patches may be nontrivial on ecological timescales, the subsequent transfer of genes may be reduced and accompanied by increased variance in reproductive success. The effect of this process in a patch–corridor framework depends on the strength of selection in each habitat patch, and whether local adaption occurs continuously along a gradient (e.g., along a corridor) or discretely within each patch. In general, where local adaption is prevalent, genetic differentiation will be higher between the patches (due to reduced gene flow) and the effect on genetic diversity will depend on the response to selection (Desai et al. 2013). Furthermore, the demographic characteristics of the species (e.g., extinction–recolonization patterns) and the rate of disturbance to the patches can affect the adaptation of populations in patch–corridor systems (Orrock 2005). In this study, we show that corridors can retain genetic diversity and decrease genetic differentiation. It is widely recognized that increased genetic diversity will aid the conservation of populations, but the benefits of maintaining reduced genetic differentiation are debatable (Frankham 2008). However, because we show that genetic differentiation and genetic diversity respond differently to corridors (e.g., Figs2 and 3A), thus conveying different information about evolutionary processes, we suggest that both genetic differentiation and genetic diversity are useful response variables for monitoring the effects of genetic drift, gene flow, and selection (Allendorf et al. 2013; Hoban et al. 2014).

This is the first study to demonstrate that corridors can reduce the negative genetic effects associated with habitat fragmentation for a wide range of species and taxa with intrinsically different population sizes and dispersal abilities. Our agent-based model is the first to demonstrate that corridors can both reduce genetic drift within and increase gene flow between subdivided populations. We further show that understanding species interactions is vital to predicting evolutionary responses to habitat fragmentation. Minimizing the negative genetic effects of habitat fragmentation will better serve populations of any species to respond to changing environmental conditions. From a management perspective, the benefits provided by increased genetic resilience will have to be carefully weighed against any potential negative ecological effects of corridors (Haddad et al. 2014; Resasco et al. 2014). Understanding that corridors can reduce the negative genetic effects of habitat fragmentation for entire communities has important conservation and management implications: determining how to best apply these principals in practice will be the next big conservation challenge.

## Data archiving statement

Data available from the Dryad Digital Repository: http://dx.doi.org/10.5061/dryad.88h07

## Supporting Information

**Figure S1.** Response of genetic diversity in the patches to corridor length (*x*-axis) and corridor width (colored circles) for each of the four species groups. **Figure S2.** Response of genetic differentiation (calculated from individuals sampled within the patches) to corridor length (*x*-axis) and corridor width (colored circles) for each of the four species groups. **Figure S3.** Average genetic differentiation for each species group across all tested corridor designs. **Figure S4.** The relationship between genetic diversity and genetic differentiation for different corridor configurations. **Figure S5.** A heat map illustrating the difference in genetic diversity between scenarios without increased corridor mortality and those with high (90%) corridor mortality. **Figure S6.** Response of genetic differentiation to corridor length (*x*-axis) and corridor width (colored circles) for each of the four species groups after corridor mortality was increased to 90%. **Figure S7.** Response of genetic differentiation (*F*_ST_ ; panels A & B ) and genetic diversity (Number of unique genotypes; panels C & D) to increasing corridor mortality.
