# Supplementary material for: Habitat corridors facilitate genetic resilience irrespective of species dispersal abilities or population sizes
Source: Evol Appl. 2015 Mar 31;8(5):454–63. doi: 10.1111/eva.12255 (PMC4430769; doi:10.1111/eva.12255)

**Supporting Figures**

**Fig. S1:** Response of genetic diversity in the patches to corridor length (x-axis) and corridor width (colored circles) for each of the four species groups. Both corridor width and corridor length were measured in patch lengths (see Fig. 1) and genetic diversity was measured as the total number of unique genotypes in all habitat patches.


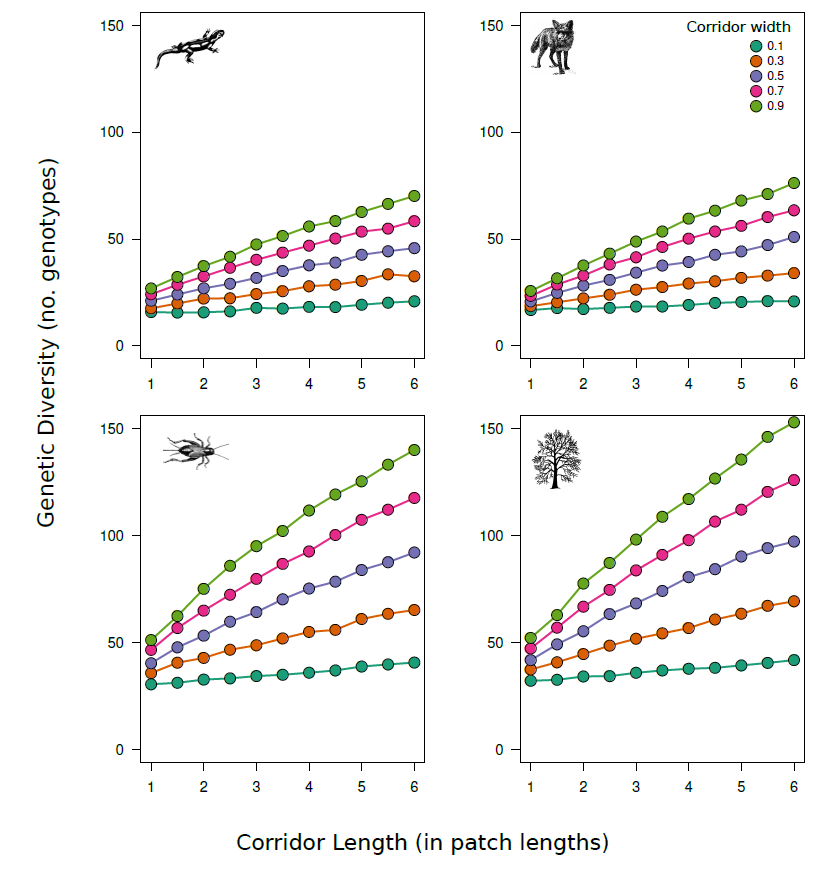


**Fig. S2:** Response of genetic differentiation (calculated from individuals sampled within the patches) to corridor length (x-axis) and corridor width (colored circles) for each of the four species groups. Both corridor width and corridor length were measured in patch lengths. Notice that even small increases in corridor width (e.g., from 0.1 to 0.3), can result in large reductions in genetic differentiation between habitat patches.


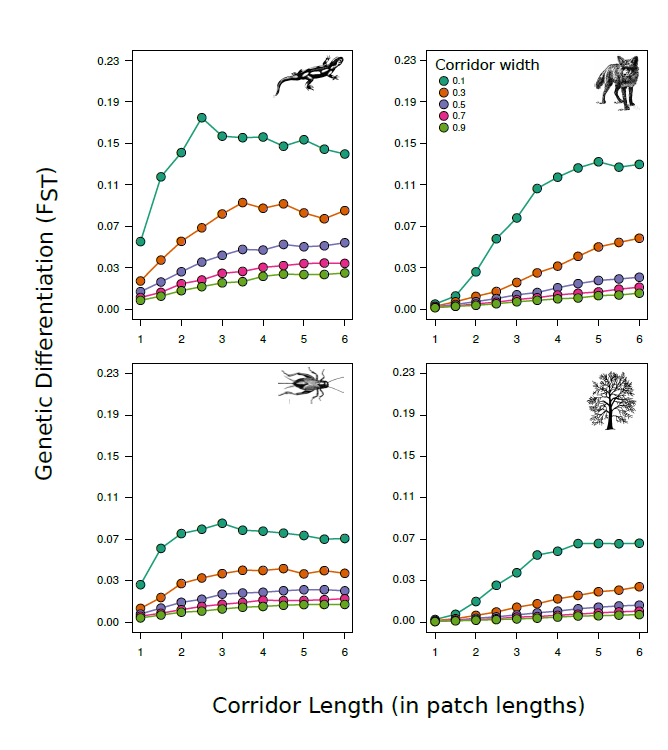


**Fig. S3:** Average genetic differentiation for each species group across all tested corridor designs. Filled circles equal the mean and error bars represent the 95% confidence intervals. Two model types were explored: one where habitat sizes in the patches was held constant and the area of corridors was varied (‘variable area’) and another where the total area of the patches and corridors was held constant such that if corridor area was increased, then the area of the habitat patches was decreased by an equivalent amount (‘fixed area’). For both cases the qualitative, but not quantitative, results were similar. Notice that species with small population sizes and short dispersal distances (salamander) had higher levels of genetic differentiation than species with larger population sizes and far dispersal distances (tree).


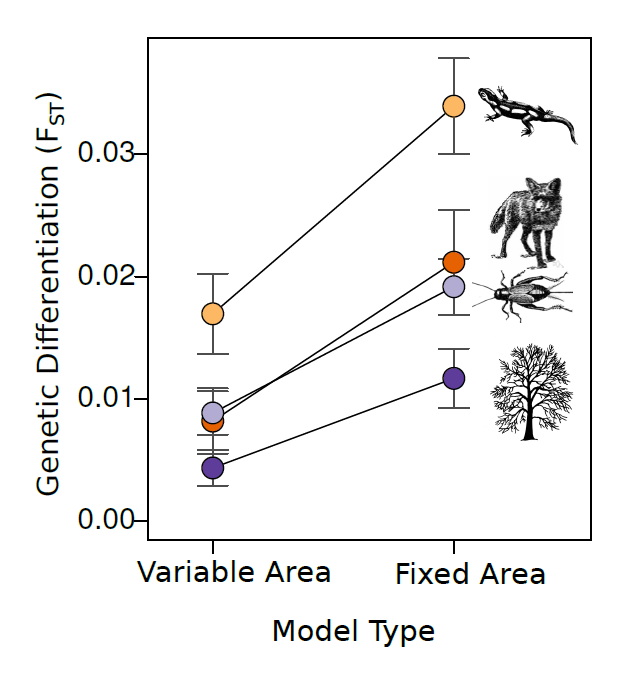


**Fig. S4:** The relationship between genetic diversity and genetic differentiation for different corridor configurations. For a given corridor width (shown as different colors; see legend in top-left panel), each increase along the x and y axes (line segments) corresponds to an increase in corridor length. Even small increases in corridor width can result in substantial decreases in *F_ST_* with concomitant increases in genetic diversity and this general relationship is maintained irrespective of whether the life history characteristics of a taxon is more similar to a tree as opposed to a salamander.


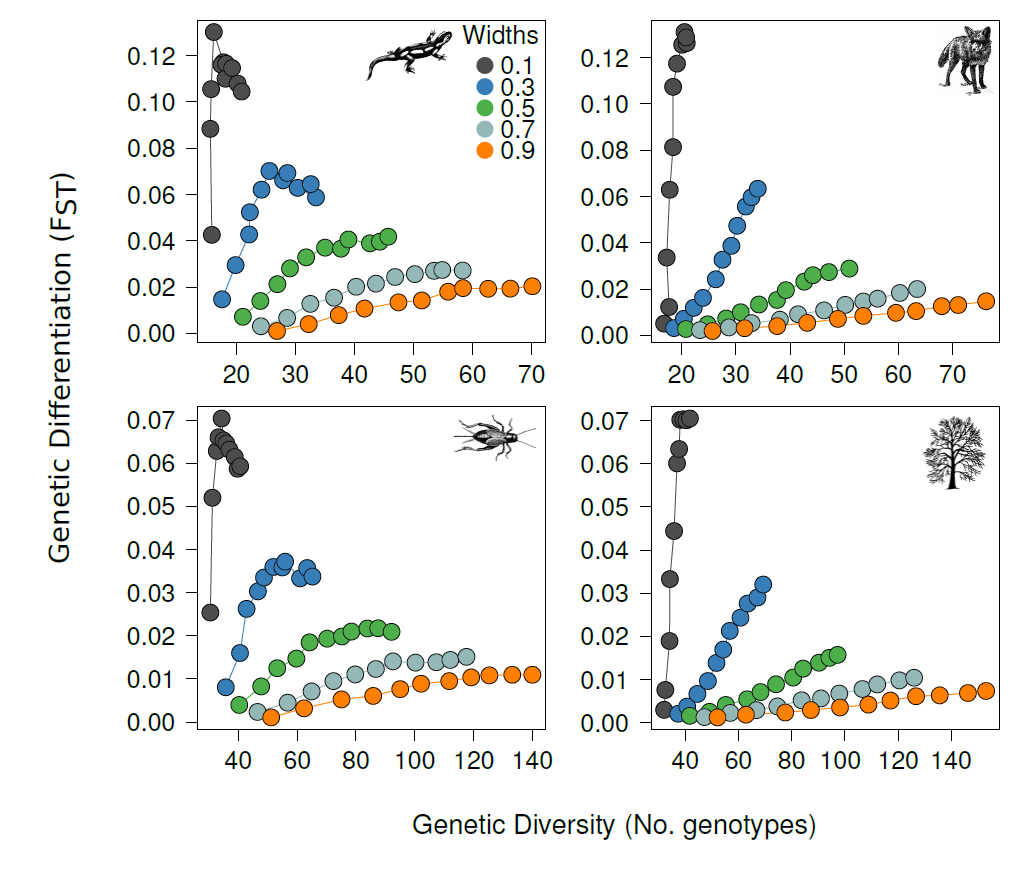


**Fig. S5:** A heat map illustrating the difference in genetic diversity between scenarios without increased corridor mortality and those with high (90%) corridor mortality. Species with large population sizes experienced the largest decreases in genetic differentiation (panels C and D). Notice that in many cases, corridor mortality resulted in a decrease in genetic diversity, which suggests that effective habitat corridors (those with low mortality) may be the most useful for mitigating the negative genetic effects of habitat fragmentation. For all species groups, there was not much of a reduction in genetic diversity at small corridor widths because genetic diversity was already low in models with no corridor mortality. The nearly symmetrical changes from top right to lower left reflect the proportional contributions of corridor length and width to genetic diversity (see Fig. 1*A*, Fig. S1).


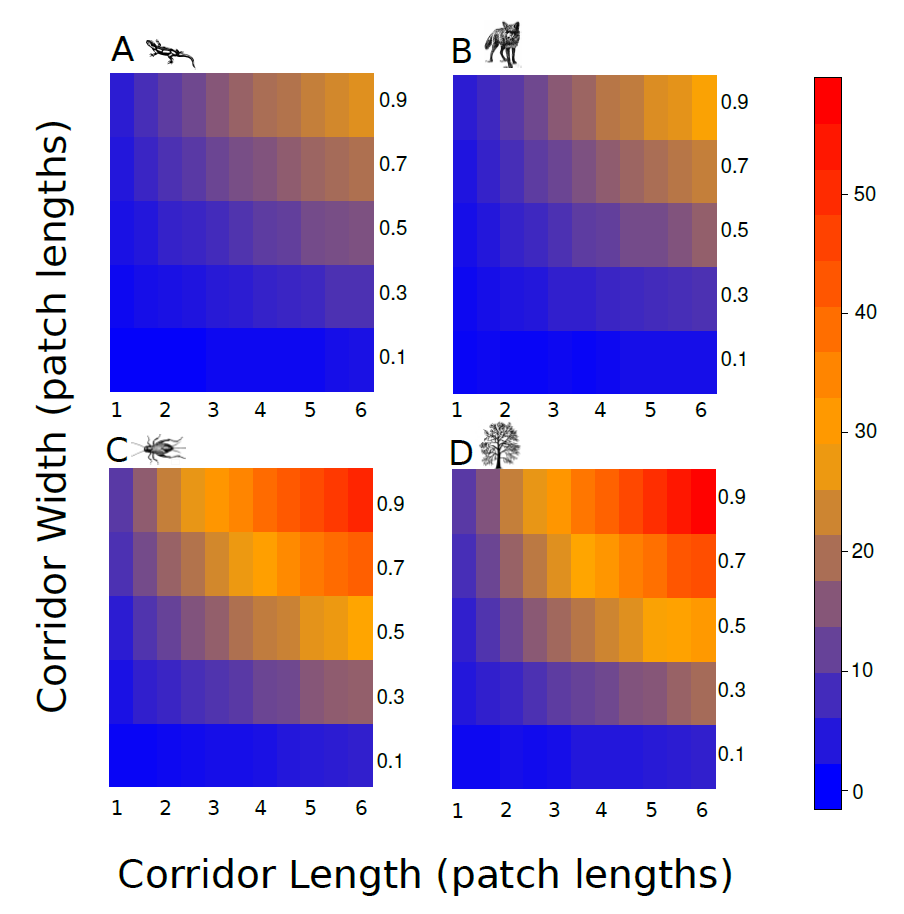


**Average Reduction in Genetic Diversity (No. Genotypes)**

**Fig. S6:** Response of genetic differentiation to corridor length (x-axis) and corridor width (colored circles) for each of the four species groups after corridor mortality was increased to 90%. Notice that the benefits of increasing corridor width are much reduced in this model when compared to models with little or no corridor mortality (*c.f.* Fig. S2).


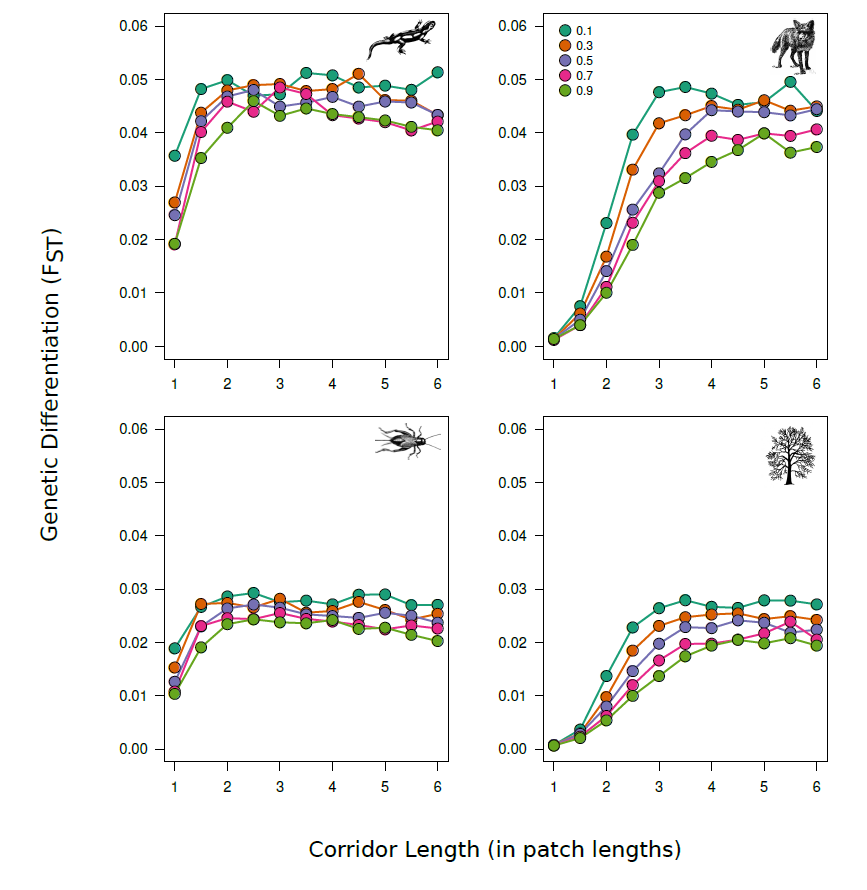


**Fig. S7:** Response of genetic differentiation (*F_ST_* ; panels A & B ) and genetic diversity (Number of unique genotypes; panels C & D) to increasing corridor mortality. Genetic differentiation and diversity were measured from individuals sampled within habitat patches and are plotted for corridors with the median corridor width and length (panels A & C), and the smallest corridor width and longest corridor length (B & D). As corridor mortality increases, genetic diversity decreases linearly, while *F_ST_* increases at an increasing rate. Notice, however, that even patches connected by corridors with high mortality (e.g., 90%) would fare better than patches with no corridors at all.


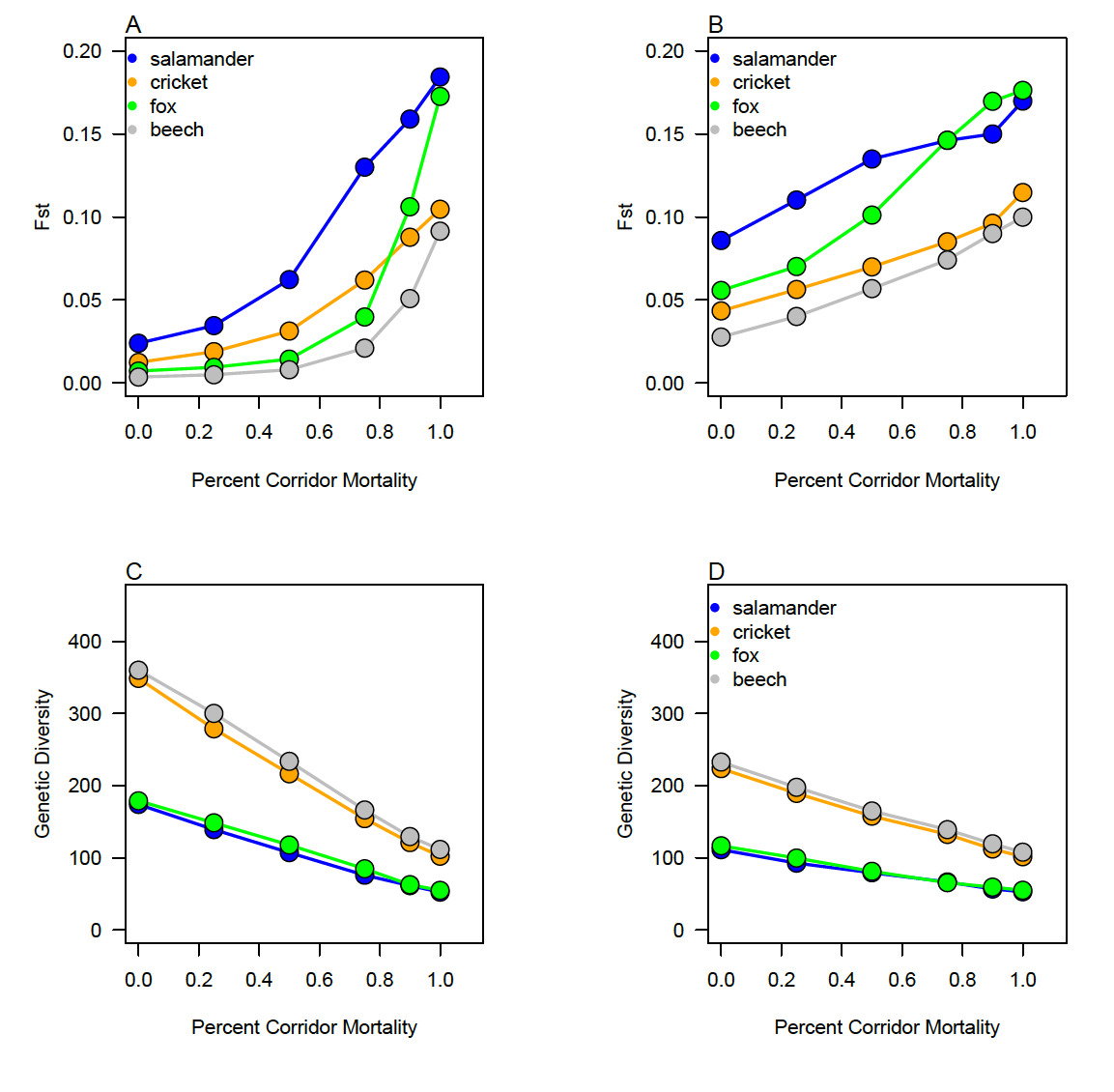

Supplement: Supplementary file 1 — Figure S1. Response of genetic diversity in the patches to corridor length (x-axis) and corridor width (colored circles) for each of the four species groups. Figure S2. Response of genetic differentiation (calculated from individuals sampled within the patches) to corridor length (x-axis) and corridor width (colored circles) for each of the four species groups. Figure S3. Average genetic differentiation for each species group across all tested corridor designs. Figure S4. The relationship between genetic diversity and genetic differentiation for different corridor configurations. Figure S5. A heat map illustrating the difference in genetic diversity between scenarios without increased corridor mortality and those with high (90%) corridor mortality. Figure S6. Response of genetic differentiation to corridor length (x-axis) and corridor width (colored circles) for each of the four species groups after corridor mortality was increased to 90%. Figure S7. Response of genetic differentiation (FST ; panels A & B ) and genetic diversity (Number of unique genotypes; panels C & D) to increasing corridor mortality. [file eva0008-0454-sd1.docx]
